# Food Ingredient Extracts of *Cyclopia subternata* (Honeybush): Variation in Phenolic Composition and Antioxidant Capacity

**DOI:** 10.3390/molecules171214602

**Published:** 2012-12-07

**Authors:** Dalene de Beer, Alexandra E. Schulze, Elizabeth Joubert, André de Villiers, Christiaan J. Malherbe, Maria A. Stander

**Affiliations:** 1 Post-Harvest and Wine Technology Division, Agricultural Research Council (ARC), Infruitec-Nietvoorbij, Private Bag X5026, Stellenbosch, 7599, South Africa; E-Mails: joubertL@arc.agric.za (E.J.); malherbech@arc.agric.za (C.J.M.); 2 Department of Food Science, Stellenbosch University, Private Bag X1, Matieland (Stellenbosch), 7602, South Africa; E-Mail: 15477479@sun.ac.za; 3 Department of Chemistry and Polymer Science, Stellenbosch University, Private Bag X1, Matieland (Stellenbosch), 7602, South Africa; E-Mail: ajdevill@sun.ac.za; 4 Department of Biochemistry, Stellenbosch University, Private Bag X1, Matieland (Stellenbosch), 7602, South Africa; E-Mail: lcms@sun.ac.za

**Keywords:** antioxidant, *Cyclopia subternata*, food ingredient, phenolic compounds, quality

## Abstract

*Cyclopia subternata* plants are traditionally used for the production of the South African herbal tea, honeybush, and recently as aqueous extracts for the food industry. A *C. subternata* aqueous extract and mangiferin (a major constituent) are known to have anti-diabetic properties. Variation in phenolic composition and antioxidant capacity is expected due to cultivation largely from seedlings, having implications for extract standardization and quality control. Aqueous extracts from 64 seedlings of the same age, cultivated under the same environmental conditions, were analyzed for individual compound content, total polyphenol (TP) content and total antioxidant capacity (TAC) in a number of assays. An HPLC method was developed and validated to allow quantification of xanthones (mangiferin, isomangiferin), flavanones (hesperidin, eriocitrin), a flavone (scolymoside), a benzophenone (iriflophenone-3-*C*-β-glucoside) and dihydrochalcones (phloretin-3',5'-di-*C*-β-glucoside, 3-hydroxyphloretin-3',5'-di-*C*-hexoside). Additional compounds were tentatively identified using mass spectrometric detection, with the presence of the 3-hydroxyphloretin-glycoside, an iriflophenone-di-*O*,*C*-hexoside, an eriodictyol-di-*C*-hexoside and vicenin-2 being demonstrated for the first time. Variability of the individual phenolic compound contents was generally higher than that of the TP content and TAC values. Among the phenolic compounds, scolymoside, hesperidin and iriflophenone-3-*C*-β-glucoside contents were the most variable. A combination of the measured parameters could be useful in product standardization by providing a basis for specifying minimum levels.

## 1. Introduction

The use of plant extracts as functional ingredients in food has increased substantially during recent years. Antioxidant extracts remain a major segment of the functional ingredient market as oxidative stress is an underlying factor in many disease conditions [1]. Therefore, new antioxidant ingredients or new sources of known antioxidant ingredients are the subject of many scientific investigations [2]. One such source is *Cyclopia subternata* Vogel (Family: Fabaceae; Tribe: Podalrieae), an endemic South African fynbos plant that traditionally has been used as a herbal tea called honeybush after “fermentation”, a high temperature oxidative process required to produce its characteristic sweet aroma and flavor. Mostly ignored in terms of its commercial potential in the previous century, commercial production of *C. subternata* commenced in the 1990s to meet the demand for honeybush by the local South African and international markets [3].

In step with the global focus on phenolic compounds of plant extracts as important health-promoting constituents, investigation of the phenolic composition of *C. subternata* showed the presence of the xanthones, mangiferin and isomangiferin, as major constituents [4,5,6]. As herbal tea and food ingredient extract it contributes these compounds to the diet [7]. An aqueous extract of *C. subternata* was shown to have anti-diabetic properties [8]. The health-promoting properties of mangiferin have been documented, ranging, amongst others, from antioxidant [9], anti-inflammatory [10], anti-diabetic [11] and hypolipidemic [11] to enhancement of recognition memory [12]. This has led to the development of standardized extracts from mango stem bark and leaves (*Mangifera indica* L.; Family: Anacardiaceae), both considered to be good sources of mangiferin [13,14]. Ethanol and aqueous extracts from mango leaf contain, respectively, 7.3% and 1.0% mangiferin [13]. The reported decrease in mangiferin content and antioxidant activity of honeybush extracts, due to the traditional fermentation process [15,16], prompted the introduction of green honeybush (*i.e.*, plant material cut and dried without fermentation) to the market. Previous studies, using a small number of samples (n = 6 for each study) showed aqueous extracts from green *C. subternata* to contain 1.19% and 2.73% mangiferin [15,16], indicating that this plant is worth further investigation as source material for preparation of a standardized extract.

Other antioxidant phenolic compounds of interest that are present in relatively high quantities in aqueous extracts of *C. subternata* include the flavanones hesperidin and eriocitrin, and the flavone scolymoside [15]. Hesperidin and eriocitrin, as citrus flavonoids, have received attention for their health-promoting properties [17,18,19,20,21]. Scolymoside, better known as a constituent of artichoke [22], is an aldose reductase inhibitor [23]. By comparison, its radical scavenging activity was found to be better than that of chlorogenic acid [24]. Recently, Kokotkiewicz *et al*. [6] showed the presence of iriflophenone-3-*C*-β-glucoside, a benzophenone and precursor of mangiferin, and the dihydrochalcone phloretin-3',5'-di-*C*-β-glucoside in *C. subternata.* Both compounds, by nature of their chemical structures, could contribute to the antioxidant activity of extracts of *C. subternata*. 

Commercial propagation and cultivation of *C. subternata* are mostly limited to the use of seedlings. The seeds are randomly collected from natural populations and from plantations established with seeds from natural populations. Large variation in phenolic content is thus to be expected due to a high level of genetic variation. A previous study by our group, undertaken to determine the extent of variation of mangiferin content in green *C. subternata* plant material, demonstrated values ranging between 0.06% and 3.11%, with the lowest values obtained for stems and the highest for leaves [25]. The purpose of the present study was to determine the variation in the phenolic composition of hot water extracts prepared from a large number of green *C. subternata* seedling plants, as well as the impact of this variation on the total antioxidant capacity. Hot water extracts were chosen as they are relevant for the food and supplement industry. For this purpose a high-performance liquid chromatography-diode array detection (HPLC-DAD) method for quantification of the major phenolic compounds in the extracts was developed and validated. Liquid chromatography-mass spectrometry (LC-MS) and -tandem mass spectrometry (LC-MS/MS) were also used for the tentative identification of new phenolic compounds in the analyzed extracts.

## 2. Results and Discussion

### 2.1. HPLC-DAD Method Development

The HPLC method previously described by de Beer and Joubert [15] to quantify the major phenolic compounds in *C. subternata* suffered from a few limitations, including the co-elution of unidentified compound(s) with isomangiferin and a complicated integration process due to the elution of eriocitrin, scolymoside and phloretin-3',5'-di-*C*-β-glucoside on a polymeric hump. This method was therefore adapted in the current contribution to improve its applicability to analysis of *C. subternata* extracts.

Four columns were evaluated with 1% acetic acid, 2% acetic acid or 0.1% formic acid as aqueous phase and acetonitrile as organic modifier, using the gradient as described by De Beer and Joubert [15]. The performances of the columns were evaluated with respect to problematic separation areas in the chromatograms of randomly chosen green and fermented *C. subternata* extracts (n = 1 each). The Gemini NX and the Kinetex columns provided the best potential separation of mangiferin and isomangiferin from the unidentified co-eluting compounds, resulting in the selection of these two columns to proceed with method development. Methanol was subsequently evaluated as organic modifier after adjusting the gradient to accommodate the differences in solvent strength between methanol and acetonitrile. Methanol did not provide better separation than acetonitrile on either column, and resulted in maximum pressures above the instrumental limit (P_max_ = 400 bar) for the Kinetex column. Thus the Gemini NX column and acetonitrile were selected for further optimization.

Of the three acidified aqueous phases evaluated, 2% acetic acid provided the best separation, especially for mangiferin and isomangiferin, from the unidentified compounds. Subsequent evaluation continued with 2% acetic acid and acetonitrile as mobile phases. The gradient was changed to a scouting gradient from 10% to 40% acetonitrile to remove the smaller steps in the previously employed gradient [15]. This approach spread out the polymeric hump previously observed, while maintaining good separation of the major phenolic compounds and simplifying integration. In order to improve separation around mangiferin and isomangiferin the initial acetonitrile content was decreased to 8%. Furthermore, gradient times from 25 to 35 min in one min intervals were evaluated. Use gradient times longer than 25 min did not improve separation and a 25 min gradient was thus selected as optimal. Temperature evaluation was performed in the range of 25 to 50 °C with 30 °C leading to the lowest degree of co-elution of hesperidin, eriocitrin and iriflophenone-3-*C*-β-glucoside. Similarly, mangiferin and isomangiferin also displayed the best resolution at this temperature.

### 2.2. Identification of Phenolic Compounds in C. subternata

**Figure 1:**
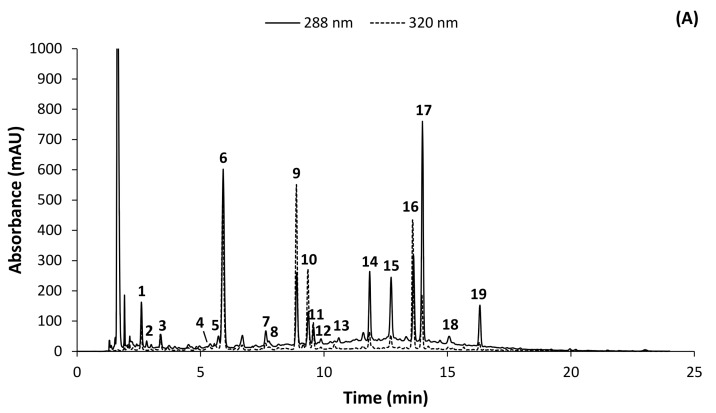

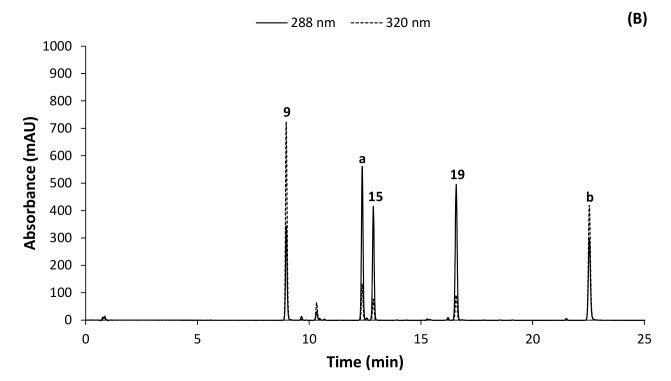
Chromatograms showing (**A**) phenolic compounds identified in freeze-dried aqueous extracts of green *Cyclopia subternata* and (**B**) a standard calibration mixture [see Table 1 for identity of numbered peaks; **a** aspalathin; **b** luteolin].

**Figure 2:**
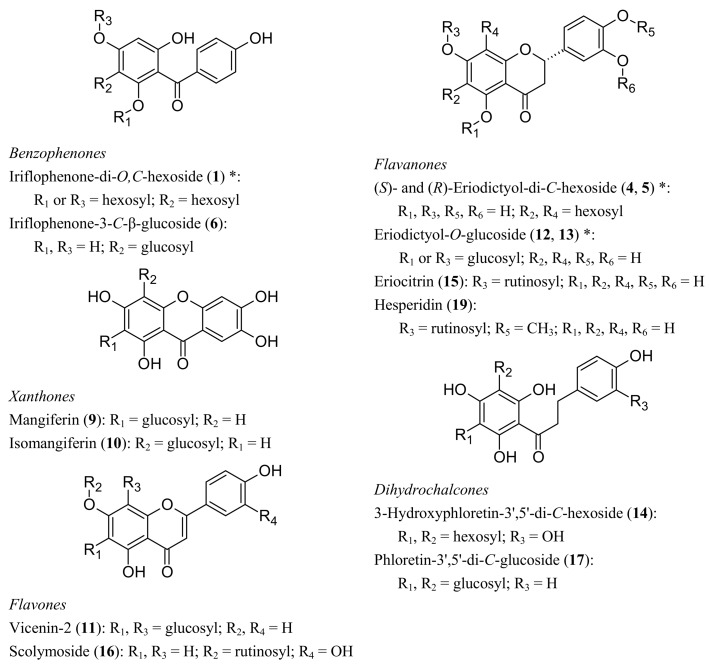
Structures of phenolic compounds identified in freeze-dried aqueous extracts of green and fermented *Cyclopia subternata* (* indicates that the position of glycoside moieties are not certain).

Liquid-chromatography with mass spectrometric (LC-MS) and tandem mass spectrometric (LC-MS/MS) analyses of one extract each of green and fermented *C. subternata* were performed in both positive and negative ionization modes. By comparing retention times, UV-Vis spectral properties, LC-MS spectra and LC-MS/MS fragmentation patterns with those of authentic commercial standards (Figure 1 and Table 1), the presence of mangiferin (**9**), eriocitrin (**15**), and hesperidin (**19**) were confirmed in the *C. subternata* extracts. The presence of isomangiferin (**10**) was confirmed by comparison with isolated isomangiferin [4]. Non-standard compounds were tentatively identified (Table 1) by comparison of UV-Vis, LC-MS and LC-MS/MS data with previous research literature. These compounds are discussed in more detail for the different phenolic classes below. Structures for known and tentatively identified compounds are shown in Figure 2.

#### 2.2.1. MS/MS Fragmentation of *C. subternata* Phenolics

*Cyclopia subternata* flavonoids include both flavonoid *O-* and *C-*glycosides, which can relatively easily be distinguished based on their divergent mass spectral properties. Fragmentation of *O*-glycosides at low collision energies typically involves cleavage of the *O*-glycosidic bond, with corresponding losses of 162, 146 and 132 amu for hexoses, deoxyhexoses and pentoses, respectively [26]. *C-*glycosides, on the other hand, do not contain labile bonds; therefore higher collision energies are required for fragmentation. Under these conditions most of the fragmentation involves the sugar moiety. Losses of 90, 96, 120 and 150 amu are typical for hexoses, and 60, 90 and 120 amu for pentoses, respectively. These are often accompanied by additional loss of water molecules (−18 amu). In the case of flavonoid-di-*C-*glycosides, simultaneous fragmentation of both sugars is common, leading to a relatively large number of fragments typically being detected (for example [M−H-96-120-H_2_O]^−^ for a flavonoid-di-*C-*hexoside). 

#### 2.2.2. Benzophenones

Compound **6** with *m/z* 407 ([M−H]^−^) was detected at a retention time of 6.69 min and has the proposed molecular formula C_19_H_19_O_10_ [M−H]^−^. The compound presented a major fragment ion at *m/z* 287 corresponding to [M−H-120]^−^, which indicates a *C*-glycoside structure. Additionally, other fragments including *m/z* 317 and 257, corresponding to losses of [M−H−90]^−^ and [M−H−150]^−^, respectively, are also characteristic of *C-*glycosides. Based on the UV-Vis spectral data, MS and MS/MS fragmentation patterns, this compound was identified as iriflophenone-3-*C-*β*-*glucoside, which has previously been isolated and described by Kokotkiewicz *et al.* [6]. Peak **1** with *m/z* 569 ([M−H]^−^) was detected at a retention time of 3.10 min and has the proposed molecular formula of C_25_H_29_O_15_ ([M−H]^−^). The compound presented several fragment ions: *m/z* 479 [M−H−90]^−^, *m/z* 449 [M−H−120]^−^, *m/z* 317 [M−H−90−162]^−^, *m/z* 287 [M−H−120−162]^−^. This fragmentation pattern possibly indicates a phenolic compound containing 2 hexosyl groups, of which one is a *C-*hexosyl and the other a *O-*hexosyl. This compound also showed similar characteristics to iriflophenone-*C-*β*-*glucoside (*m/z* 407, [M−H]^−^), according to its UV and MS/MS spectra. Due to its mass difference of 162 amu, compound **1** was proposed to be a glycosylated derivative of iriflophenone-*C-*β*-*glucoside. Its earlier elution time also corresponds to the glycosylated derivative. Therefore compound **1** has tentatively been identified as an iriflophenone-di-*C*,*O-*hexoside.

#### 2.2.3. Dihydrochalcones

Compound **17** with *m/z* 597 ([M−H]^−^) eluted at 13.53 min and has a proposed molecular formula C_27_H_33_O_15_ ([M−H]^−^). This molecule displayed MS/MS fragments at *m/z* 477 ([M−H−120]^−^), 459 ([M−H−120−H_2_O]^−^), 417 ([M−H−2 × 90]^−^), 387 ([M−H−120−90]^−^) and 357 ([M−H−2 × 120]^−^), which correspond with the dihydrochalcone phloretin-3',5'-di-*C-*β*-*glucoside previously identified in *C. subternata* [6]. The UV spectrum of this compound is also in agreement with literature [6].

**Figure 3:**
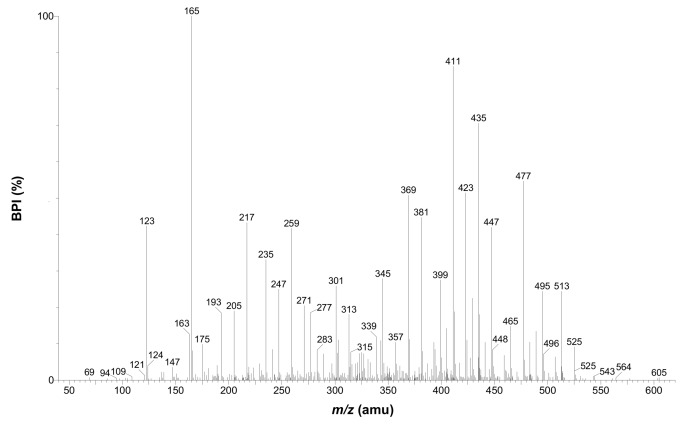
LC**-**MS/MS spectrum of 3-hydroxyphloretin-3',5'-di-*C*-β-hexosyl (**14**) obtained in positive ionization mode.

Compound **14**, which eluted at 11.54 min with a *m/z* of 613 ([M−H]^−^) and 615 ([M−H]^+^), had a UV spectrum and MS/MS fragmentation pattern similar to that of phloretin-3',5'-di-*C-*β*-*glucoside (**17**, *m/z* 597 [M−H]^−^), but displayed a molecular ion 16 amu higher than that of **17**. This indicates a possible hydroxylated derivative of phloretin-3',5'-di-*C-*β*-*glucoside. Compound **14** has the proposed molecular formula C_27_H_35_O_16_ ([M−H]^+^), which was in good agreement with its accurate mass, 615.1927 ([M+H]^+^). In fact, these data are in accordance with UV, MS and MS/MS data of a compound previously reported in rooibos tea, *Aspalathus linearis* [27]. The following fragments were observed for compound **14** in positive ionization mode: *m/z* 525 ([M+H−90]^+^), *m/z* 495 ([M+H−120]^+^), *m/z* 477 ([M+H−120−H_2_O]^+^), *m/z* 465 ([M+H−150]^+^), *m/z* 447 ([M+H−150−H_2_O]^+^), *m/z* 435 ([M+H−2 × 90]^+^), *m/z* 423 ([M+H−2 × 96]^+^), *m/z* 411 ([M+H−90−96−H_2_O]^+^), *m/z* 399 ([M+H−96−120]^+^), *m/z* 381 ([M+H−96−120−H_2_O]^+^), *m/z* 369 ([M+H−96−150]^+^), *m/z* 345 ([M+H−120−150]^+^) and *m/z* 327 ([M+H−120−150−H_2_O]^+^) (Figure 3). This fragmentation pattern is identical to that of the novel C-5'-hexosyl derivative of aspalathin (2',3,4,4',6',-pentahydroxy-3',5'-di-*C*-hexosyldihydrochalcone) reported by Beelders *et al*. [27,28]. This compound is therefore the 3-hydroxy-derivative of phloretin-3',5'-di-*C-*β*-*glucoside (**17**). This proposition is entirely consistent with the mass difference of 16 amu observed between **14** and **17**, while the fragments detected at *m/z* 123 and 165 in the MS/MS spectrum of the former confirm that the additional hydroxyl group is attached to the B-ring. Compound **14** will be referred to as 3-hydroxyphloretin-3',5'-di-*C*-hexoside. It is interesting to note that the related compound, aspalathin (2',3,4,4',6'-pentahydroxy-3'-*C*-β-D-glucopyranosyldihydrochalcone), considered to be a unique constituent of rooibos tea, was not observed in any of the *C. subternata* extracts analyzed. To confirm that compound **14** is the same molecule previously observed in rooibos, a rooibos extract was analyzed using the current *C. subternata* HPLC method*.* Identical retention times and MS data confirmed this was indeed the same compound (results not shown).

#### 2.2.4. Flavone and Flavanone *C*-glycosides

Compounds **4** and **5** were characterized by molecular ions at *m/z* 611 ([M−H]^−^, *t_R_* = 6.32 and 6.50 min, respectively) and an identical proposed molecular formula of C_27_H_31_O_16_ ([M−H]^−^). Both compounds showed numerous fragments under MS/MS conditions, including *m/z* 491 ([M−H−120]^−^), 431 ([M−H−2 × 90]^−^), 401 ([M−H−90−120]^−^) and 371 ([M−H−2 × 120]^−^). These fragments indicate a di-*C-*hexoside, which together with their molecular weights and formulae point to an eriodictyol-di-*C-*hexoside. The known reversed phase elution order allows tentative identification of compounds **4** and **5** as (*S*)-eriodictyol-di-*C*-hexoside and (*R*)-eriodictyol-di-*C*-hexoside, respectively [27,29].

Compound **11** with a retention time of 9.40 min and *m/z* of 593 [M−H]^−^ and 595 [M+H]^+^ has tentatively been identified as apigenin-6,8-di-*C-*glucoside (vicenin-2) with the proposed molecular formula C_27_H_29_O_15_ [M−H]^−^. This compound had a UV spectrum corresponding to that of a flavone derivative, with maximum absorbance at 272 and 331 nm. MS/MS fragmentation showed the following fragments: *m/z* 505 ([M−H−90]^+^), *m/z* 457 ([M−H−120−H_2_O]^+^), *m/z* 427 ([M−H−150−H_2_O]^+^), *m/z* 409 ([M−H−90−96]^+^), *m/z* 379 ([M−H−120−96]^+^), *m/z* 355 ([M−H−2 × 120]^+^), *m/z* 337 ([M−H−2 × 120−H_2_O]^+^) and *m/z* 325 ([M−H−150−120]^+^). These data are in accordance with literature reports for apigenin-6,8-di-*C-*glucoside detected in *A. linearis* [27,30].

#### 2.2.5. Flavone and Flavanone *O*-Glucosides

Compounds **12** and **13** were detected at retention times of 10.04 and 10.63 min, respectively, and both displayed molecular ions at *m/z* of 449 ([M−H]^−^) and UV spectra characteristic of flavanones. MS/MS data showed fragments at *m/z* 287 ([M−H−162]^−^) and *m/z* 151 for both molecules. Based on these data, compounds **12** and **13** were tentatively identified as eriodictyol-*O-*glucoside isomers. These compounds are believed to be isomers of one another, as one displayed a higher intensity of the *m/z* 287 ion and the other of the *m/z* 151 ion (Table 1). Eriodictyol-5-*O*-glucoside and -7-*O*-glucoside have previously been isolated from the related species, *Cyclopia intermedia*, and identified by nuclear magnetic resonance (NMR) spectroscopy [31]. Assignment of peaks **12** and **13** as one or the other of these isomers was however not possible based on relative retention or MS/MS spectral information. Alternatively, these compounds may also be the (*R*)- and (*S*)-diastereomers of eriodictyol-5-*O*-glucoside, which, in contrast to the corresponding diastereomers of eriodictyol-7-*O*-glucoside, can be separated by RP-LC [32].

Compound **16** exhibited a molecular ion at *m/z* 593 [M−H]^−^ at a retention time of 13.29 min. UV and MS/MS spectra of this compound corresponded to those of scolymoside (luteolin-7-*O*-rutinoside). A major fragmentation product was observed at *m/z* 285 [M−H−308]^−^, which is consistent with the loss of rutinoside. The presence of scolymoside has previously been established in *C. subternata* [5,6].

#### 2.2.6. Additional Unidentified Phenolic Compounds

Several additional compounds could not be identified based on the available data, although UV-Vis and MS data point to their phenolic nature. These compounds together with their relevant UV, MS and MS/MS data are listed in Table 2. Further research is required to determine the identity of these molecules. Compounds **8** and **18** may possibly be naringenin derivatives, but due to the lack of literature examples of MS/MS data, they remain unidentified.

### 2.3. HPLC-DAD Method Validation

Method validation was performed in order to determine the reliability of the method for routine analyses of *C. subternata* samples. A fermented *C. subternata* extract was included in the validation in order to confirm that the method is compatible with extracts prepared from both green and fermented *C. subternata*. The method was deemed specific for the quantified peaks, as their UV-Vis and MS spectra matched those of authentic reference standards or literature values as described in the previous section.

The linearity of the calibration curves was excellent (Table 3). All y-intercepts were fairly low and the Pearson’s product moment correlation coefficients were all equal to 1.000. The stability of all compounds in the standard calibration mixtures and the reconstituted *C. subternata* extracts was very good over a 26-h-period (Table 4). The % change for all the compounds ranged from −3.5% to 1.4%, except for 3-hydroxyphloretin-3',5'-di-*C-*hexoside (5.5% for unfermented extract; 9.1% for fermented extract). The % relative standard deviation (RSD) of all the compounds over the six time-points was also less than 3%. This indicated that no substantial decrease in any of the compounds occurred during this period. Ascorbic acid was added to standard calibration mixtures and samples, as it proved to be crucial to ensure stability of phenolic compounds, especially dihydrochalcones, in rooibos infusions [33]. Similarly, the intra- and inter-day precision of the method was very good, with the RSD for all the compounds in the standard calibration mixtures and the reconstituted *C. subternata* extracts less than 2% (Table 5).

### 2.4. Differences in Phenolic Composition and TAC of Aqueous Extracts of C. subternata Leaves and Stems

*Cyclopia subternata*, with a typical lifespan of seven to eight years, is harvested annually by cutting all growth 30 to 50 cm above the ground to stimulate development of new shoots. However, if not harvested regularly, thick stems develop. The shoots of different bushes, varying in leaf size, leaf density and stem thickness, are cut into small pieces and dried for production of green honeybush [3]. For its use as herbal tea the processed plant material is sieved to remove the coarse stem fraction, but for extract production the plant material is used without sieving. In order to evaluate the effect of varying stem-to-leaf ratio on the phenolic composition and TAC of aqueous extracts of green *C. subternata*, extracts were prepared from separated stems and leaves of selected bushes (n = 10).

**Figure 4:**
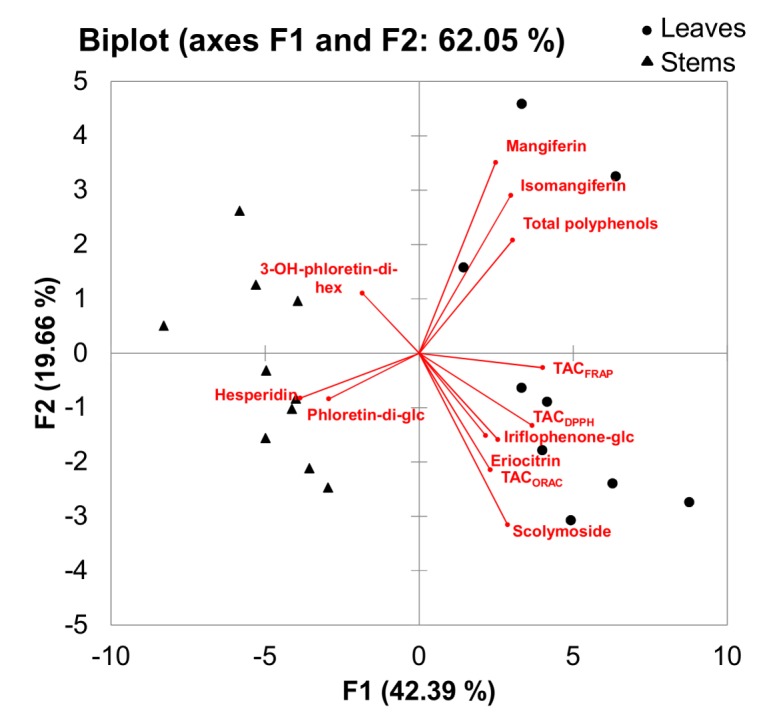
Principal component biplot for phenolic composition and total antioxidant capacity (TAC) of freeze-dried aqueous extracts of green *Cyclopia subternata* leaves (n = 10) and stems (n = 10).

*Abbreviations*: iriflophenone-glc, iriflophenone-3-*C*-β-glucoside; phloretin-di-glc, phloretin-3',5'-di-*C*-β-glucoside; 3-OH-phloretin-di-hex, 3-hydroxy-phloretin-3',5'-di-*C*-hexoside].

Extract yield was significantly affected by the plant part used for extraction, with the average value for leaves double that of the stems (Table 6). The phenolic composition of aqueous stem and leaf extracts was qualitatively similar, but the content of all compounds, except 3-hydroxyphloretin-3',5'-di-*C-*hexoside, differed significantly (*p* < 0.05) between the two types of extracts. Leaf extracts had more than twice the mangiferin, isomangiferin and scolymoside contents than the stem extracts (*p* < 0.05). In addition, the contents of iriflophenone-3-*C*-β*-*glucoside and eriocitrin (eriodictyol-7-*O*-rutinoside) were 55% and 22%, respectively, higher in leaf extracts compared to stem extracts (*p* < 0.05). The stem extracts, on the other hand, contained 3.1 times more of the flavanone hesperidin and 1.4 times more of the dihydrochalcone phloretin-3',5'-di-*C*-β*-*glucoside compared to the leaf extracts. The differences in phenolic composition resulted in moderately higher (1.2 times; *p* < 0.05) total polyphenol and TAC_DPPH_ and TAC_FRAP_ values, while the TAC_ORAC_ values were not affected (*p* ≥ 0.05). Higher stem-to-leaf ratio will, therefore, affect the phenolic composition and TAC of aqueous green *C. subternata* extracts. These differences are highlighted in a PCA biplot (Figure 4) with all stem extracts associated with hesperidin, phloretin-3',5'-di-*C*-β*-*glucoside and 3-hydroxyphloretin-3',5'-di-*C-*hexoside contents, while the leaf extracts were associated with the other measured parameters.

Manufacturing of extracts from leaves only would be more economical due to a higher yield, although separation of leaves and stems may not be feasible. The use of only leaves would also yield an extract high in xanthones, iriflophenone-3-*C*-β*-*glucoside, scolymoside and eriocitrin with good antioxidant capacity values. Stem extracts, on the other hand, could be a valuable source of hesperidin and would be preferred if an extract high in hesperidin is the aim. Even after taking yield into account, the stems are a better source of hesperidin.

### 2.5. Variation in Phenolic Composition and TAC of Aqueous Extracts from C. subternata Seedlings from Combined Leaf and Stem Material

**Figure 5:**
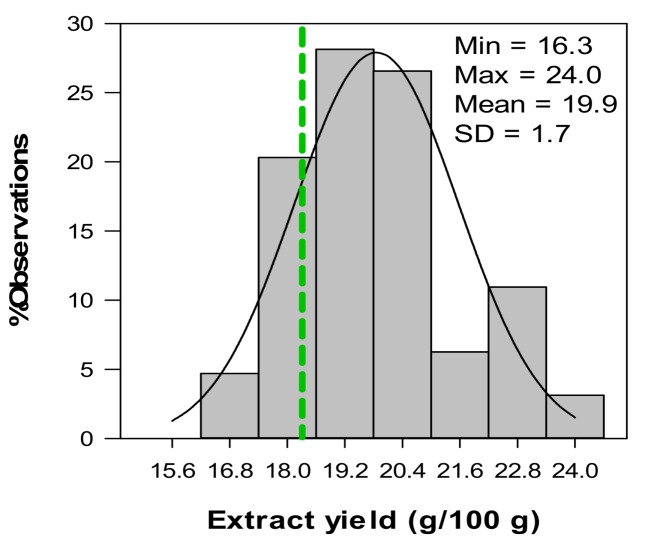
Distribution of the aqueous extract yield obtained for green *Cyclopia subternata* (n = 64) [dotted green line indicates the 20% quintile].

Extract yield for aqueous extracts from *C. subternata* seedlings varied between 16.3% and 24.0% (Figure 5). Extract yield values for plant material (consisting of leaves and stems) were similar or lower than for leaves only. A low extract yield has negative implications for the economic feasibility of extract production. A potential factor affecting extract yield is the stem-to-leaf ratio of the plant material as discussed in the previous section. 

**Figure 6:**
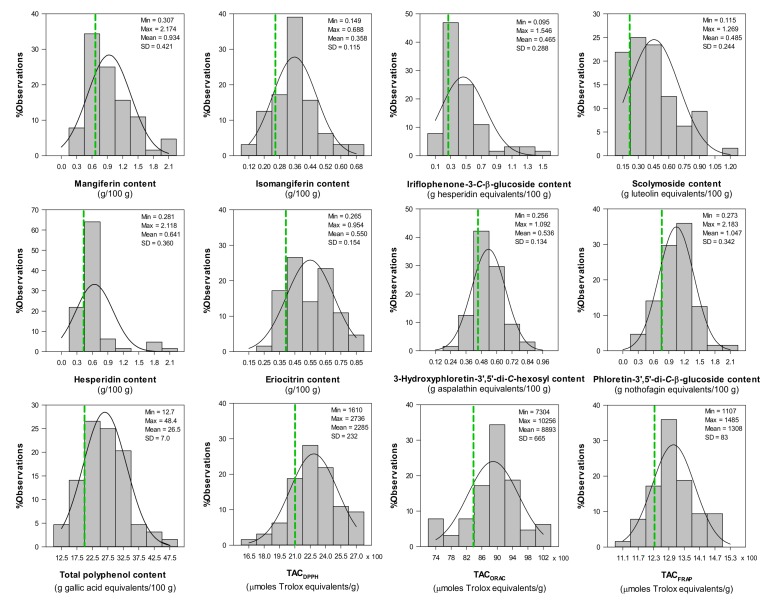
Distribution of phenolic composition and total antioxidant capacity (TAC) of freeze-dried aqueous extracts of green *Cyclopia subternata* (n = 64) [dotted green line indicates the 20% quantile].

The phenolic composition of aqueous extracts from *C. subternata* seedlings (Figure 6) was comparable to previous reports for such extracts [15,16], taking into account subsequent identification of compounds, noted as unidentified in previous reports, by Kokotkiewicz *et al*. [6]. The highest mean values were observed for the phloretin-3',5'-di-*C*-β*-*glucoside (1.05%) and mangiferin (0.9%) contents, while values higher than 0.5% were also observed for hesperidin (0.64%), eriocitrin (0.55%) and 3-hydroxyphloretin-3',5'-di-*C-*hexoside (0.54%) contents. All other content values were below 0.5%, with isomangiferin content (0.36%) the lowest. The total polyphenol content, expressed as gallic acid equivalents, had a mean value (26.5%) lower than that reported previously (32.4%) [16], but within the range found for the leaf extracts (Table 6). The phenolic composition of the extracts varied considerably. %RSD, indicating the variability of the parameter, was >40% for mangiferin (45%), iriflophenone-3-*C*-β*-*glucoside (62%), scolymoside (50%) and hesperidin (56%) contents, while the content of the other compounds and the total polyphenol content were less variable (%RSD < 34%).

The TAC of extracts was determined using three commonly used assays, namely the DPPH^•^ scavenging, ORAC and FRAP assays. The DPPH^•^ scavenging assay was chosen as it employs a stable radical, is easy to execute and is already used by South African herbal extract producers as a quality control parameter [34]. The ORAC assay, on the other hand, is often used for products destined for the American market [35], while the FRAP assay gives an indication of iron reducing ability, in contrast to the radical scavenging activity measured by the other two assays. The aqueous extracts of *C. subternata* seedlings showed mean TAC_DPPH_, TAC_ORAC_ and TAC_FRAP_ values of 2285, 8893 and 1308 µmoles Trolox equivalents/g. The TAC_DPPH_ and TAC_FRAP_ values were similar to those previously obtained using the ABTS radical cation scavenging assay [16]. The TAC_DPPH_ and TAC_ORAC_ values were also similar to those obtained for aqueous extracts of fermented rooibos prepared in a similar manner [34]. Rooibos is well-known for its antioxidant activity [36]. TAC values for all assays were much less variable (%RSD 6%–10%) than individual compound contents.

Two potential causes of variation in phenolic composition and TAC exist for the dataset under consideration. As the seedlings were all of the same age and were harvested at the same time in the same plantation, variation due to season, climatic conditions, soil properties and geographical location were considered negligible. Chemotypic variation between seedlings and variation in stem-to-leaf ratio between seedlings are both possible. If the stem-to-leaf ratio was the dominant factor negative correlations between compounds found in higher concentration in the leaves and compounds found in higher concentration in the stems would be expected. However, no such correlations were observed (e.g., *r* = −0.135, *p* = 0.287 for mangiferin and hesperidin content). Genetic variation between seedlings is, therefore, the most probable cause of variation in phenolic composition and TAC of aqueous extracts of *C. subternata* seedlings. Phenolic composition is known to be very variable between different genotypes of a plant species, e.g., apple [37]. When considering the ratios between maximum and minimum values for the content of individual compounds, the variation in *C. subternata* extracts were much higher than reported for fermented rooibos aqueous extracts also originating from seedling plant material [34]. The rooibos extracts were, however, prepared from different production batches consisting of pooled material from a large number of plants. Pooling of material from different plants is expected to decrease the variation between batches.

The highly variable nature of aqueous extracts of *C. subternata* seedlings has serious implications for product standardization. The 20% quintile calculated for the dataset may be used as a starting point for determining minimum levels for quality control of extracts. Selecting a parameter that is too variable or setting a minimum level for quality control purposes may lead to very high batch fail rates. On the other hand, parameters that support the intended function of the product should be selected. The results suggest that TAC values would be more suitable as quality control parameters than individual compound contents. If standardization needs to be performed using a single compound or combination of compounds, the use of selected, vegetatively propagated plant material is recommended.

## 3. Experimental

### 3.1. Chemicals

All chemicals were analytical grade and sourced from Sigma-Aldrich (St. Louis, MO, USA) or Merck Chemicals (Darmstadt, Germany), unless otherwise specified. Authentic reference standards with purity >95% were obtained from Sigma-Aldrich [hesperidin, gallic acid, (±)-6-hydroxy-2,5,7,8-tetramethylchromane-2-carboxylic acid (Trolox)] and Extrasynthese (Genay, France; mangiferin, eriocitrin, luteolin). Aspalathin (>95%) and nothofagin (phloretin-3'-*C*-glucoside) (>95%) were obtained from PROMEC (Medical Research Council of South Africa, Tygerberg, South Africa). HPLC gradient grade acetonitrile was purchased from Merck. Deionized water, prepared using an Elix (Millipore, Milford, MA, USA) water purification system, was further purified to HPLC grade using a Milli-Q Academic (Millipore) water purification system.

### 3.2. Sourcing of Plant Material

*Cyclopia subternata* Vogel (Family: Fabaceae; Tribe: Podalrieae) plant material, consisting of leaves and stems (shoots), was harvested from individual seedling plants of the same age (n = 64) at Kanetberg Flora (Barrydale district, South Africa). The freshly harvested *C. subternata* shoots were dried intact at 40 °C in a temperature-controlled drying tunnel with forced air circulation to ca. 8%–10% moisture content and ground with a Retsch mill (1 mm sieve; Retsch GmbH, Haan, Germany). These samples represented green *C. subternata*. Ten samples, consisting of either leaves or stems were also prepared by separating the leaves from the stems after drying, before grinding them separately. One green and one fermented *C. subternata* sample were prepared from the same bush for method validation. The leaves and stems were shredded and one half dried immediately as described above and then sieved (1.4 mm). The other half was “fermented” as follows: the cut plant material was moistened to ca. 60%–65% moisture content and fermented at 90 °C for 16 h, followed by drying and sieving as described for green *C. subternata*.

### 3.3. Preparation of Aqueous Extracts

An extract of each sample was prepared by adding boiling water (70 mL) to the milled plant material (7 g) in a screw-cap glass bottle, which was placed in a water bath at 93 °C for 30 min. The mixture was stirred every 5 min. The resulting extract was filtered through Whatman #4 filter paper while warm, followed by cooling to room temperature in a water bath. The volume of the recovered extracts was measured using a volumetric cylinder. The soluble solids content of the extracts were determined gravimetrically after evaporation of the water on a steam bath (ca. 1 h) followed by drying in a forced-air circulating oven (100 °C for 1 h). The extract yield was calculated as the amount of soluble solids in the recovered extract volume as a percentage of the extracted plant material. Extracts were frozen and freeze-dried using a VirTis Advantage Plus freeze-drier (SP Scientific, Warminster, PA, USA).

### 3.4. HPLC-DAD Method Development and Validation

#### 3.4.1. Method Development

Analyses were conducted on an Agilent 1200 series HPLC instrument which consisted of an in-line degasser, quaternary pump, autosampler, column oven and DAD, controlled by Chemstation software (Agilent Technologies Inc., Santa Clara, CA). The solvent gradient described by de Beer and Joubert [15] was evaluated on four different columns in order to improve separation of co-eluting compounds. The Zorbax Eclipse XDB-C18 (150 × 4.6 mm, 5 µm, 80 Å) and Zorbax SB-C18 (100 × 4.6 mm; 1.8 µm) columns from Agilent Technologies (Waldbronn, Germany), as well as the Kinetex C18 (150 × 4.6 mm; 2.6 µm; 100 Å) and Gemini-NX C18 (150 × 4.6 mm; 3 µm; 110 Å) columns from Phenomenex (Santa Clara, CA, USA), were tested.

The mobile phase initially consisted of 2% acetic acid (A) and acetonitrile (B). One or both mobile phases was also replaced by 1% acetic acid or 0.1% formic acid and methanol, respectively, and evaluated. A gradient from 8 to 38% B was evaluated at different temperatures ranging from 25 to 50 °C (5 °C intervals) and different gradient times from 25 to 30 min (1 min intervals) using 2% acetic acid and acetonitrile as mobile phases.

#### 3.4.2. Quantification of Individual Phenolic Compounds using HPLC-DAD

Dimethylsulfoxide (DMSO) was used to prepare stock solutions of standards and aliquots were frozen at −20 °C until analysis. Extracts were dissolved in purified water (ca. 6 mg/mL) and frozen at −20 °C until analysis. Ascorbic acid (ca. 9 mg/mL final concentration) was added to standard mixtures and defrosted reconstituted extracts, where after the mixtures were filtered using 0.22 µm pore-size Millex-HV syringe filters with 4 and 33 mm diameters (Millipore), respectively. Ascorbic acid was added to prevent oxidative degradation of the phenolic compounds. The injection volume for extracts was 15 µL and for the standards 10–20 µL. The Gemini-NX C18 (150 × 4.6 mm; 3 µm; 110 Å) column was selected for the quantification method, with 2% acetic acid (A) and acetonitrile (B) as mobile phases. Separation was carried out at 30 °C with the following mobile phase gradient at a flow rate of 1 mL/min: 0–2 min (8% B), 2–27 min (8%–38% B), 27–28 min (38%–50%), 28–29 min (50% B), 29–30 min (50%–8% B), 30–40 min (8% B). UV-Vis spectra were recorded for all samples from 200 to 550 nm. The xanthones (mangiferin and isomangiferin) and flavone (scolymoside) were quantified at 320 nm, and the flavanones (eriocitrin and hesperidin), benzophenone (iriflophenone-3-*C*-glucoside) and dihydrochalcones (phloretin-3',5'-di-*C*-β-glucoside and 3-hydroxyphloretin-3',5'-di-*C*-hexoside) at 288 nm. A seven-point calibration curve was set up for all the available authentic standards, as well as standards needed to calculate equivalent values. Scolymoside and iriflophenone-3-*C*-glucoside were quantified as luteolin and hesperidin equivalents, respectively, as no authentic reference standards were available. Isomangiferin was quantified using a response factor previously determined for isomangiferin relative to mangiferin, while phloretin-3',5'-di-*C*-β-glucoside and 3-hydroxyphloretin-3',5'-di-*C*-hexoside were quantified as phloretin-3'-*C*-β-glucoside (nothofagin) and 3-hydroxyphloretin-3'-*C*-β-glucoside (aspalathin) equivalents, respectively, using a response factor previously determined for nothofagin relative to aspalathin. The standard calibration mixtures accommodated the expected concentrations present in the sample extracts with the following ranges (given in µg compound on-column): mangiferin (0.032–2.889 µg), hesperidin (0.009–2.263 µg), luteolin (0.005–1.360 µg), eriocitrin (0.007–1.689 µg), and aspalathin (0.007–1.660 µg).

#### 3.4.3. Identification of Phenolic Compounds using LC-DAD-MS and -MS/MS Detection

LC-DAD-MS and -MS/MS analyses were conducted on a Waters Acquity UPLC equipped with a binary pump, in-line degasser, autosampler, column oven and DAD detector (Waters, Milford, MA, USA). The system was coupled to a Synapt G2 Q-TOF system (Waters) equipped with an electrospray ionization (ESI) source. The same method was used as described in Section 3.4.2, however, the entire gradient was delayed by 0.2 min. Furthermore, for negative ESI, solvent B was also increased with 0.5% at each interval of the gradient, and the column temperature increased to 32 °C. These changes were required to obtain similar separation as on the Agilent HPLC system. An injection volume of 3 µL was used for both the standard and samples. UV-Vis spectra were recorded from 235 to 450 nm. The eluent was split 3:2 prior to introduction into the ionization chamber. MS data were acquired in both positive and negative ionization mode. Two analyses were performed for each sample in each ionization mode: in the first analysis, MS and MS^E^ data were acquired (the latter using a collision energy ramp from 25 to 60 V); subsequently, a second analysis was performed to acquire MS/MS data using a collision energy of 30 V. The MS parameters were as follows: desolvation temperature, 275 °C; nitrogen flow rate, 650 L/h; source temperature, 120 °C; capillary voltage, 2,500 V; cone voltage, 15 V. Data acquired were processed using MassLynx v.4.1 software (Waters). Peaks were identified by comparison of retention times, UV-Vis spectra and LC-MS spectra to those of authentic standards. LC-MS and -MS/MS spectra were compared to literature to identify or tentatively identify peaks for which no authentic standards were available.

#### 3.4.4. Method Validation

One standard calibration mixture injected at two injection volumes to represent a low and intermediate point in the calibration curve and one of each of the fermented and green *C. subternata* freeze-dried aqueous extracts were chosen for method validation. Specificity of the method was determined by evaluating the UV-Vis and MS spectra of peaks selected for quantification. Linearity of the response was determined by calculating the slope, y-intercept and correlation coefficient (r) of each calibration curve. Stability of the standard calibration mixture and samples was determined by injecting them repeatedly over a 26-h-period. The %RSD and % change over the period were determined for each of the compounds over the set interval. Intra-day precision was determined by consecutive repeated injections of the standard mixture and samples six times on the same day. The %RSD was calculated for each of the compounds. The same procedure was repeated over three days, and compared by calculating the %RSD for the averaged values for each day to determine the inter-day precision.

### 3.5. Determination of Total Phenol Content and Total Antioxidant Capacity (TAC)

A BioTek Synergy HT microplate reader equipped with Gen5 software for data acquisition (Winooski, VT, USA) was employed for absorbance and fluorescence readings. Total phenol content of the extracts was determined in triplicate using the Folin-Ciocalteau method adapted for 96-well microplates [38]. The TAC of the infusions was determined in triplicate using the DPPH radical scavenging [38], ORAC [34] and FRAP assays in 96-well microplate format.

### 3.6. Statistical Analysis

Data for leaf (n = 10) and stem (n = 10) extracts were subjected to univariate analysis of variance (ANOVA) using SAS, version 9.2 (SAS Institute, Cary, NC, USA). The Shapiro-Wilk test was performed to test for normality. Student’s t test was used to calculate the least significant difference (LSD) at the 5% level to facilitate a comparison of mean values. Principal component analysis (PCA) was performed using XLSTAT software (Version 7.5.2, Addinsoft, New York, NY, USA). Calculations for construction of distribution plots, Kolmogorov-Smirnov analysis for normality and Pearson’s product moment correlation analysis for aqueous extracts from *C. subternata* seedlings (n = 64) were performed using SAS. 

## 4. Conclusions

Honeybush (*Cyclopia* spp.) food ingredient extracts are popular for production of ready-to-drink iced teas and other food products. *Cyclopia subternata*, as one of the few cultivated *Cyclopia* spp., is a good source for production of aqueous extracts, containing a variety of phenolic compounds including xanthones, dihydrochalcones, flavanones, flavones and benzophenones. Marketing of such extracts can focus on the content of specific compounds with health-promoting benefits or on the bioactivity of the extracts, e.g., anti-diabetic properties. Standardization of extracts is becoming more and more important in the food ingredient industry. Cultivation from seedlings with a high level of genetic variation and subsequent high variation in individual compound contents as shown for a large number of samples, impede standardization. Total polyphenol content and TAC may be considered as alternative quality parameters for the first tier of standardization as they vary to a lesser extent. In future, cultivation of selected, vegetatively propagated plant material could facilitate product standardization. To our knowledge four compounds, namely iriflophenone-di-*O*,*C*-hexoside, (*R*)- and (*S*)-eriodictyol-di-*C*-hexoside, vicenin-2 and 3-hydroxyphloretin-3',5'-di-*C*-hexoside, were tentatively identified in *C. subternata* for the first time.

## Figures and Tables

**Table 1:** UV-Vis, LC-MS and LC-MS/MS characteristics of phenolic compounds identified in freeze-dried aqueous extracts of green and fermented *Cyclopia subternata*.

Peak ^a^	Mode	t_R_ (min)	Accurate mass^b^	λ_max_ (nm)	Error (ppm)	Proposed molecular formula	Fragments	Phenolic compound
**1**	+	3.10	571.1664	290	0.2	C_25_H_31_O_15_	373, 355, 337, 325, 313, 289, 271, 259, 231, 219, 195 *, 177, 165	Iriflophenone-di-*O*,*C*-hexoside
	−		569.1488		−2.3	C_25_H_29_O_15_	479, 449, 317, 287 *	
**4**	+	6.32	613.1780	285	1.8	C_27_H_33_O_16_	475, 409, 339, 327, 303, 285, 261 *, 219	(*S*)-Eriodictyol-di-*C*-hexoside
	−		611.1621		1.0	C_27_H_31_O_16_	491, 431, 401, 371 *	
**5**	+	6.50	613.1780	285	1.8	C_27_H_33_O_16_	475, 409, 339, 327, 303, 285, 261 *, 219	(*R*)-Eriodictyol-di-*C*-hexoside
	−		611.1621		1.0	C_27_H_31_O_16_	491, 431, 401, 371 *	
**6**	+	6.69	409.1136	294	0.2	C_19_H_21_O_10_	391, 289, 231, 195 *, 177, 165, 121	Iriflophenone-3-*C*-β-glucoside
	−		407.0981		1.0	C_19_H_19_O_10_	317, 287 *, 257, 245, 215, 201, 193, 165, 125	
**9**	+	8.95	423.0920	234, 257, 317, 366	−1.7	C_19_H_19_O_11_	351, 339, 327, 303, 299, 285, 273 *, 257	Mangiferin
	−		421.077		−0.2	C_19_H_17_O_11_	331, 301 *, 271, 259	
**10**	+	9.30	423.0922	234, 255, 316, 366	−1.2	C_19_H_19_O_11_	405, 357, 341, 327, 303 *, 299, 287, 285, 273, 261	Isomangiferin
	−		421.0769		−0.5	C_19_H_17_O_11_	331, 301 *, 273, 271, 259	
**11**	+	9.40	595.1671	235, 270, 331	1.3	C_27_H_31_O_15_	505, 457, 427, 421, 409, 391, 379, 355, 337, 325 *, 307, 295	Apigenin-6,8-di-*C*-glucoside [Vicenin-2]
	−		593.1499		−1.2	C_27_H_29_O_15_	503, 473 *, 383, 353	
**12**	+	10.04	451.1229	281	−2.4	C_21_H_23_O_11_	289, 163, 153 *	Eriodictyol-*O*-glucoside
	−		449.1069		−3.3	C_21_H_21_O_11_	287, 151 *, 135	
**13**	+	10.63	451.1232	281	−1.8	C_21_H_23_O_11_	289 *, 163, 153	Eriodictyol-*O*-glucoside
	−		449.1080		−0.9	C_21_H_21_O_11_	287, 151 *, 135, 107	
**14**	+	11.54	615.1927	283	0.3	C_27_H_35_O_16_	525, 495, 477, 465, 447, 435, 423, 411, 399, 381, 369, 345, 327, 259, 247, 235, 217, 205, 165 *, 123	3-Hydroxyphloretin-3',5'-di-*C*-hexoside
	−		613.1772		0.5	C_27_H_33_O_16_	493, 475, 433, 403, 373 *, 361, 331, 239, 209	
**15**	+	12.50	597.1812	283	−1.2	C_27_H_33_O_15_	289 *, 273, 153	Eriodictyol-7-*O*-rutinoside [Eriocitrin]
	−		595.1661		−0.3	C_27_H_31_O_15_	287 *, 151, 135	
**16**	+	13.29	595.1647	252, 348	−2.7	C_27_H_31_O_15_	449, 287 *	Luteolin-7-*O*-rutinoside [Scolymoside]
	−		593.1509		0.5	C_27_H_29_O_15_	285 *	
**17**	+	13.53	599.1972	284	−0.7	C_27_H_35_O_15_	479, 461, 449, 431, 419, 407, 395 *, 383, 365, 353, 329, 301, 107	Phloretin-3',5'-di-*C-*β*-*glucoside
	−		597.1831		2.0	C_27_H_33_O_15_	477, 459, 417, 387, 357 *, 345, 315	
**19**	+	15.85	611.197	283	−1.0	C_28_H_35_O_15_	449, 303 *, 177, 153	Hesperetin-7-*O*-rutinoside [Hesperidin]
	−		609.1837		3.0	C_28_H_33_O_15_	301 *	

^a^ Peak numbers correspond to numbered peaks in Figure 1; ^b^ accurate mass determined experimentally; * ion with highest relative intensity.

**Table 2:** UV-Vis, LC-MS and LC-MS/MS characteristics of unidentified compounds in freeze-dried aqueous extracts of green and fermented *Cyclopia subternata*.

Peak ^a^	Mode	t_R_ (min)	Accurate mass ^b^	λ_max_ (nm)	Error (ppm)	Proposed molecular formula	Fragments	Proposed identity
**2**	+	3.20	345.1186	234, 272	−0.3	C_15_H_21_O_9_	123 *,165	Unknown
	−		343.1024		−1.5	C_15_H_19_O_9_	163, 119 *	
**3**	+	4.20	425.1084	234, 315	0.2	C_19_H_21_O_11_	261, 243, 231, 219, 195 *, 177, 165, 137, 121	Unknown
	−		423.0929		0.5	C_19_H_19_O_11_	333, 303, 223, 193 *, 165, 151, 109	
**7**	+	7.83	nd	282	nd	nd	n.d.	Unknown
	−		457.1353		1.5	C_20_H_25_O_12_	163 *, 119	
**8**	+	7.93	597.1812	280	−1.2	C_27_H_33_O_15_	405, 393, 363, 339, 327, 321, 285, 273, 261 *, 219, 207	Naringenin-di-*C*-hexoside
	−		595.1665		0.3	C_27_H_31_O_15_	475, 415, 385 *, 355	
**18**	+	14.68	581.1837	279	−5.7	C_27_H_33_O_14_	273 *, 153	Naringenin-*O*-dihexoside
	−		579.1719		0.9	C_27_H_31_O_14_	271 *, 151	

^a^ Peak numbers correspond to numbered peaks in Figure 1; ^b^ accurate mass determined experimentally; * ion with highest relative intensity; nd, not detected.

**Table 3:** Linear regression data for calibration curves.

Compound	Regression equation	*r*
Mangiferin	y = 2089.4x + 7.3	1.000
Aspalathin	y = 2351.8x + 1.0	1.000
Eriocitrin	y = 1611.6x – 0.3	1.000
Hesperidin	y = 1782.1x – 1.8	1.000
Luteolin	y = 2781.8x – 4.0	1.000

**Table 4:** Compound stability in standard calibration mixtures and freeze-dried aqueous extracts of green and fermented *Cyclopia subternata* over a 26-h-period.

Compound	%RSD (% change)			
	Calibration mixture (2 µL)	Calibration mixture (15 µL)	Unfermented extract	Fermented extract
Mangiferin	0.6 (1.4)	0.2 (0.4)	0.9 (−2.7)	1.9 (−1.7)
Aspalathin	0.8 (−0.9)	0.4 (−1.1)	-	-
Eriocitrin	0.4 (0.4)	0.1 (0.2)	0.8 (−0.2)	0.7 (−2.3)
Hesperidin	1.1 (−2.1)	0.6 (−1.8)	1.2 (−3.3)	1.0 (−3.5)
Luteolin	0.9 (−1.3)	0.4 (−1.3)	-	-
Isomangiferin	-	-	0.9 (−1.8)	1.5 (−4.4)
Iriflophenone-3-*C*-β-glucoside	-	-	1.2 (−2.3)	0.6 (−1.4)
3-Hydroxyphloretin-3',5'-di-*C*-hexoside	-	-	2.0 (5.5)	2.9 (9.1)
Scolymoside	-	-	0.9 (−2.3)	0.7 (−1.7)
Phloretin-3',5'-di-*C*-β-glucoside	-	-	0.7 (−2.0)	0.6 (−1.2)

*Abbreviations:* %RSD, % relative standard deviation.

**Table 5:** Intra- and inter-day precision (%relative standard deviation) for individual phenolic compounds as determined using standard calibration mixtures, as well as freeze-dried aqueous extracts of green and fermented *C. subternata*.

Compound	Intra-day (n = 6/day)			Inter-day (n = 3)
	Day 1	Day 2	Day 3	
*Calibration mixture (2 µL)*				
Mangiferin	0.5	0.3	0.2	1.6
Luteolin	0.3	0.6	0.5	1.2
Hesperidin	0.8	0.6	0.8	0.8
Eriocitrin	0.6	0.4	0.4	0.7
Aspalathin	0.7	0.3	0.6	1.4
*Calibration mixture (15 µL)*				
Mangiferin	0.1	0.1	0.1	1.6
Luteolin	0.1	0.1	0.4	0.8
Hesperidin	0.1	0.2	0.4	0.1
Eriocitrin	0.2	0.1	0.2	0.6
Aspalathin	0.0	0.1	0.3	1.1
*Unfermented C. subternata*				
Iriflophenone-3-*C*-β-glucoside	0.2	0.4	0.1	0.3
Mangiferin	0.1	0.1	0.1	0.1
Isomangiferin	0.1	0.1	0.1	0.2
3-Hydroxyphloretin-3',5'-di-*C*-hexoside	1.1	1.3	0.7	1.6
Eriocitrin	1.4	1.6	1.2	2.0
Scolymoside	0.1	0.1	0.2	0.3
Phloretin-3',5'-di-*C*-β-glucoside	0.1	0.2	0.2	0.1
Hesperidin	0.3	0.2	0.4	0.3
*Fermented C. subternata*				
Iriflophenone-3-*C*-β-glucoside	0.1	0.1	0.1	0.5
Mangiferin	0.2	0.4	0.7	1.5
Isomangiferin	0.2	0.1	0.1	1.2
3-Hydroxyphloretin-3',5'-di-*C*-hexoside	2.0	1.7	1.8	1.6
Eriocitrin	0.5	0.6	0.2	0.7
Scolymoside	0.2	0.3	1.0	1.2
Phloretin-3',5'-di-*C*-β-glucoside	0.2	0.8	1.0	0.4
Hesperidin	0.4	0.3	0.3	0.7

**Table 6:** Extract yield, phenolic composition and total antioxidant capacity (TAC) of freeze-dried aqueous extracts of green *Cyclopia subternata* leaves (n = 10) and stems (n = 10).

Parameter	Leaves	Stems
Extract yield ^a^	23.1 ± 2.1 (20.2–27.9) a	11.8 ± 1.8 (8.6–14.2) b
Iriflophenone-3-*C*-β-glucoside content ^b^	0.441 ± 0.131 (0.263–0.634) a	0.286 ± 0.077 (0.180–0.460) b
Mangiferin content ^a^	0.817 ± 0.359 (0.313–1.396) a	0.373 ± 0.138 (0.188–0.558) b
Isomangiferin content ^a^	0.342 ± 0.104 (0.177–0.489) a	0.156 ± 0.042 (0.099–0.223) b
3-Hydroxyphloretin-3′,5′-di-*C*-β-hexoside ^c^	0.432 ± 0.073 (0.332–0.561) a	0.493 ± 0.101 (0.311–0.597) a
Eriocitrin content ^a^	0.633 ± 0.177 (0.422–1.003) a	0.517 ± 0.127 (0.344–0.744) b
Scolymoside content ^d^	0.812 ± 0.454 (0.264–1.443) a	0.391 ± 0.242 (0.152–0.826) b
Phloretin-3′,5′-di-*C*-β-glucoside ^e^	0.899 ± 0.252 (0.631–1.364) b	1.243 ± 0.237 (0.878–1.525) a
Hesperidin content ^a^	0.504 ± 0.495 (0.147–1.517) b	1.559 ± 0.289 (1.164–1.893) a
Total polyphenol content ^f^	25.5 ± 3.7 (21.1–31.6) a	21.5 ± 2.2 (18.8–25.8) b
TAC_DPPH_^g^	2568 ± 234 (2255–2913) a	2137 ± 233 (1843–2558) b
TAC_ORAC_^g^	9445 ± 822 (7446–10479) a	8871 ± 518 (8122–9685) a
TAC_FRAP_^g^	1441 ± 74 (1309–1596) a	1236 ± 69 (1160–1374) b

Values are mean ± standard deviation (minimum–maximum) and different alphabet letters in a row indicate significant differences (*p* < 0.05) between means. ^a^ g extract/100 g plant material for extract yield and g compound/100 g extract for phenolic compound contents; ^b^ g hesperidin equivalents/100 g; ^c^ g 3-hydroxyphloretin-3'-*C*-β-glucoside equivalents/100 g; ^d^ g luteolin equivalents/100 g; ^e^ g phloretin-3'-*C*-β-glucoside equivalents/100 g; ^f^ g gallic acid equivalents/100 g; ^g^ µmoles Trolox equivalents/g. *Abbreviations:* TAC_DPPH_, total antioxidant capacity measured using the DPPH radical scavenging assay; TAC_FRAP_, total antioxidant capacity measured using the ferric reducing antioxidant power assay; TAC_ORAC_, total antioxidant capacity measured using the oxygen radical absorbance capacity assay.
